# Complicated Ventral Hernia: A Perquisite for Perforated Peptic Ulcer—Unusual Clinical Scenario

**DOI:** 10.1055/s-0041-1725158

**Published:** 2021-05-25

**Authors:** Jignesh A. Gandhi, Pravin Shinde, Bhavika Kothari, Marina Kharkongor

**Affiliations:** 1 Department of General Surgery, Seth G.S. Medical College & KEM Hospital, Parel, Mumbai, Maharashtra, India

**Keywords:** peptic ulcer, gastric ulcer perforation, irreducible ventral hernia, Graham's patch technique

## Abstract

**Introduction** Peptic ulcer usually presents to the emergency in the form of an acute abdomen, which is usually diagnosed easily either clinically or radiologically. Although its incidence has decreased with the introduction on proton pump inhibitors it is still one of the most common emergencies encountered by a surgeon.

**Case Presentation** A 60-year-old woman complained of epigastric swelling for 6 months which gradually increased and became irreducible over the last 2 months. The patient also complained of pain associated with vomiting. Radiological investigations revealed a epigastric hernia with omentum and stomach as content along with fluid collection in the right perihepatic region, with tiny air foci. The patient was explored for the same.

**Discussion** Perforated peptic ulcer is a serious complication and carries high risk of morbidity and mortality. Early diagnosis with immediate resuscitation and surgical intervention is essential to improve outcomes. This is a rare case of perforated gastric ulcer which was masked under the complicated ventral hernia.

For thousands of years, healthy people have had acute abdominal pain, nausea, vomiting, diarrhea followed by death in few hours or days. In 1670, after the sudden death of daughter of King Charles I, poisoning was suspected as the cause of death. However, autopsy revealed a hole in the anterior wall of the stomach. 1 More perforations of stomach were observed after the autopsies became more of a routine between 1600 and 1800. 2 3 The treatment of perforated peptic ulcer consists of primary closure of perforation with suture and tag of omentum on top of this. 4 5 6 7 Here, we present a case of gastric ulcer perforation with irreducible ventral hernia. The clinical features of peritonitis were absent as the perforated gastric ulcer was a content of irreducible ventral hernia which prevented patient from going into generalized peritonitis and shock. To the best of our knowledge, this is the world's first case after extensive literature research.

## Case Report

A 60-year-old housewife presented with epigastric swelling for 6 months. The swelling was progressively increasing in size over 3 months and became irreducible over the past 2 months. The patient developed pain in past 15 days associated with vomiting. The patient had approached the health care system, and was investigated, and referred to higher center for further management.

**Fig. 1:**
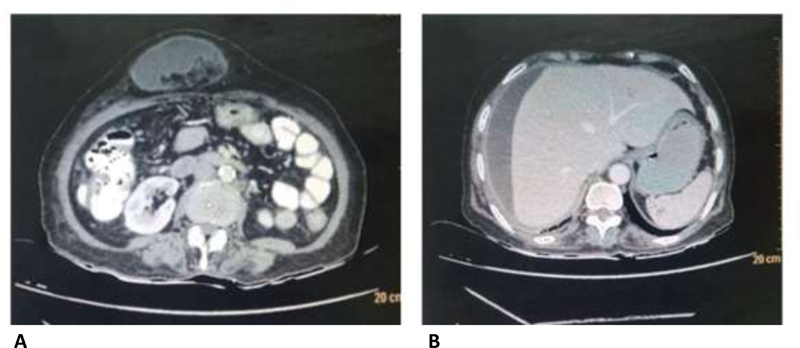
( **A** ) Epigastric hernia with fluid and omentum and transverse colon as its content ( **B** ) with right hepatic region subdiaphragmatic collection.

On examination, the patient presented with a 5 × 5 cm swelling in epigastrium, which was irreducible, tender, and with no cough impulse along with tenderness in the right hypochondrium. Blood investigations were normal. Chest X-ray and abdominal X-ray in standing position were within normal limits. Ultrasound of abdomen revealed supraumbilical hernia with trapped ascitic fluid, with perihepatic collection. A contrast-enhanced CT reported an epigastric hernia with omentum as content, along with fluid with septations and a 13 × 2 cm fluid collection in the right perihepatic region, with tiny air foci ( Fig. 1 ). The patient was taken up for surgery, as patient was symptomatic.

## Surgical Procedure

**Fig. 2:**
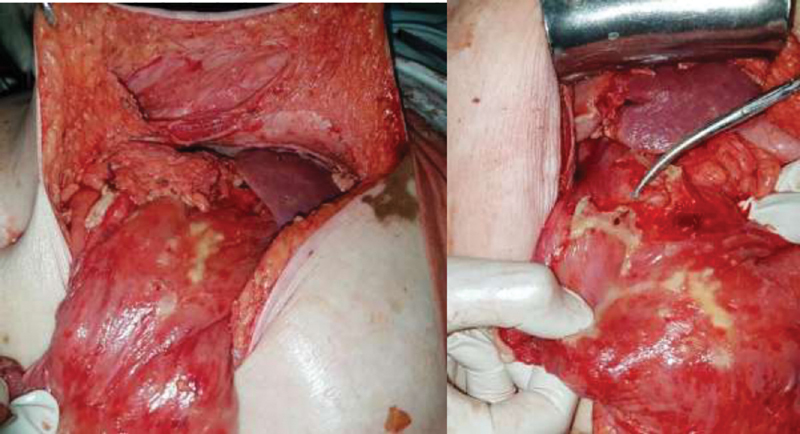
The epigastric hernia defect and prepyloric perforation.

Patient was taken up for exploratory laparotomy via upper midline incision. The hernia sac was explored, and findings were consistent with epigastric hernia with omentum and part of stomach wall as its content ( Fig. 2 ) along with frank purulent localized collection which extended into the subdiaphragmatic space on the right side. Adhesions were seen between the gall bladder, omentum, and the liver which formed a pocket in which the collection got localized. On suctioning the purulent material, a perforation of 0.5 × 0.5 cm was seen in the prepyloric region. Perforation was closed by Graham's patch technique. Anatomical repair of epigastric hernia was performed, and mesh was avoided. Postoperative period was uneventful.

## Discussion

Peptic ulcer disease affects 4 million people worldwide annually. Atypical presentation of perforated peptic ulcer occurs when the stomach is abnormal in position or when the typical gastric fluid flooding does not occur. Other indicators of toxicity may be absent in both elderly and immunocompromised patients. In our case the adhesions formed a barrier preventing the gastric secretion from tracking down into the paracolic gutters and pelvis. The presence of stomach within the hernia along with the omentum had led to further compartmentalization leading to the fluid being collected within the hernia sac and preventing generalized peritonitis.

*It became a blessing in disguise, as it prevented the patient from going into generalized peritonitis and full-blown sepsis* . Here, the condition we label as a defect (hernia) has become the almost perfect barrier for *the patient.*

## Conclusion

We have presented a case of gastric ulcer perforation, which mimicked an irreducible epigastric hernia, managed successfully by exploration and Graham's patch procedure, thus, acting as life saver to this patient despite of the *delayed presentation.*
